# Long-Term Ambient Residential Traffic–Related Exposures and Measurement Error–Adjusted Risk of Incident Lung Cancer in the Netherlands Cohort Study on Diet and Cancer

**DOI:** 10.1289/ehp.1408762

**Published:** 2015-03-27

**Authors:** Jaime E. Hart, Donna Spiegelman, Rob Beelen, Gerard Hoek, Bert Brunekreef, Leo J. Schouten, Piet van den Brandt

**Affiliations:** 1 Channing Division of Network Medicine, Department of Medicine, Brigham and Women’s Hospital and Harvard Medical School, Boston, Massachusetts, USA; 2 Exposure, Epidemiology, and Risk Program, Department of Environmental Health,; 3 Department of Epidemiology,; 4 Department of Biostatistics, and; 5 Department of Nutrition, Harvard T.H. Chan School of Public Health, Boston, Massachusetts, USA; 6 Institute for Risk Assessment Sciences, Utrecht University, Utrecht, the Netherlands; 7 Julius Center for Health Sciences and Primary Care, University Medical Center Utrecht, the Netherlands; 8 Department of Epidemiology, School for Oncology and Developmental Biology (GROW), and; 9 Department of Epidemiology, School for Public Health and Primary Care (CAPHRI), Maastricht University, Maastricht, the Netherlands

## Abstract

**Background:**

The International Agency for Research on Cancer (IARC) recently declared air pollution carcinogenic to humans. However, no study of air pollution and lung cancer to date has incorporated adjustment for exposure measurement error, and few have examined specific histological subtypes.

**Objectives:**

Our aim was to assess the association of air pollution and incident lung cancer in the Netherlands Cohort Study on Diet and Cancer and the impact of measurement error on these associations.

**Methods:**

The cohort was followed from 1986 through 2003, and 3,355 incident cases were identified. Cox proportional hazards models were used to estimate hazard ratios and 95% confidence intervals, for long-term exposures to nitrogen dioxide (NO_2_), black smoke (BS), PM_2.5_ (particulate matter with diameter ≤ 2.5 μm), and measures of roadway proximity and traffic volume, adjusted for potential confounders. Information from a previous validation study was used to correct the effect estimates for measurement error.

**Results:**

We observed elevated risks of incident lung cancer with exposure to BS [hazard ratio (HR) = 1.16; 95% CI: 1.02, 1.32, per 10 μg/m^3^], NO_2_ (HR = 1.29; 95% CI: 1.08, 1.54, per 30 μg/m^3^), PM_2.5_ (HR = 1.17; 95% CI: 0.93, 1.47, per 10 μg/m^3^), and with measures of traffic at the baseline address. The exposures were positively associated with all lung cancer subtypes. After adjustment for measurement error, the HRs increased and the 95% CIs widened [HR = 1.19 (95% CI: 1.02, 1.39) for BS and HR = 1.37 (95% CI: 0.86, 2.17) for PM_2.5_].

**Conclusions:**

These findings add support to a growing body of literature on the effects of air pollution on lung cancer. In addition, they highlight variation in measurement error by pollutant and support the implementation of measurement error corrections when possible.

**Citation:**

Hart JE, Spiegelman D, Beelen R, Hoek G, Brunekreef B, Schouten LJ, van den Brandt P. 2015. Long-term ambient residential traffic–related exposures and measurement error–adjusted risk of incident lung cancer in the Netherlands Cohort Study on Diet and Cancer. Environ Health Perspect 123:860–866; http://dx.doi.org/10.1289/ehp.1408762

## Introduction

A growing literature has demonstrated positive associations between long-term exposures to ambient air pollution and an increased risk of lung cancer. Most studies have focused on particulate matter (PM); in a recent meta-analysis including 18 studies, each 10-μg/m^3^ increase in PM ≤ 2.5 μm in diameter (PM_2.5_) was associated with a meta-relative risk of 1.09 [95% confidence interval (CI): 1.01, 1.14] (Hamra et al. 2014). However, increases in lung cancer risk have also been observed with roadway proximity and exposures to traffic-related pollutants including oxides of nitrogen (NO_2_ and NO_x_), polycyclic aromatic hydrocarbons (PAHs), and volatile organic compounds (VOCs) (Abbey et al. 1999; Beelen et al. 2008; Carey et al. 2013; Cesaroni et al. 2013; Filleul et al. 2005; Hart et al. 2011; Heinrich et al. 2013; Hystad et al. 2013; Jerrett et al. 2013; Katanoda et al. 2011; Krewski et al. 2009; Lipsett et al. 2011; Nyberg et al. 2000; Puett et al. 2014; Raaschou-Nielsen et al. 2010, 2013; Villeneuve et al. 2013, 2014; Vineis et al. 2006; Yorifuji et al. 2013). Therefore, the International Agency for Research on Cancer (IARC) recently declared ambient air pollution generally, and particulate matter specifically, carcinogenic to humans (Loomis et al. 2013).

Empirical adjustment for bias due to exposure measurement error has been applied in occupational, nutritional, and environmental epidemiology studies (Allodji et al. 2012; Armstrong 1990, 2004; Fearn et al. 2008; Heid et al. 2004; Horick et al. 2006; Keshaviah et al. 2003; Li et al. 2006; Rosner et al. 1990; Spiegelman 2010; Van Roosbroeck et al. 2008b; Zhukovsky et al. 2011). Using regression calibration, bias due to exposure measurement can be adjusted for when a validation study is available that contains information on both the standard exposure collected for the participants in the main study, as well as the “gold standard” exposure collected only in the validation study. To date, however, no study of the chronic effects of air pollution on the risk of lung cancer has incorporated adjustment for exposure measurement error.

We previously examined the associations of long-term exposures to traffic-related exposures and the risk of incident lung cancer from the Netherlands Cohort Study on Diet and Cancer (NLCS); we observed no elevations with specific pollutants, but small elevations in risk with measures of roadway proximity and traffic density (Beelen et al. 2008). Our present objective is to extend these analyses with an additional 7 years of follow-up, to determine the association of air pollution with specific histological subtypes, and to perform analyses incorporating adjustment for measurement error, using information from an exposure validation study (Van Roosbroeck et al. 2008a).

## Methods

*Study population*. Details of the NLCS population have been reported previously (Beelen et al. 2008; van den Brandt et al. 1990). Briefly, the cohort was initiated in September 1986 with 120,852 subjects 55–69 years of age living in 204 municipalities throughout the Netherlands who had not previously had cancer (other than skin cancer). All participants provided detailed information on diet, lifestyle factors, and personal characteristics at baseline. The study was designed as a case-cohort study, where cases arise over follow-up from the full cohort, but the characteristics of person-years at risk were estimated from a randomly selected subcohort of 5,000 participants. We excluded any participants from the present analysis with missing data on the exposures of interest, or on current cigarette, pipe, or cigar smoking status, resulting in a final subcohort of 4,666 members. The study was approved by the Maastricht University and the Netherlands Organization for Applied Scientific Research Institutional Review Boards and the Human Subjects Committee of the Harvard T.H. Chan School of Public Health. All cohort members consented to participate in the study by completing and returning the self-administered questionnaire.

*Outcome assessment*. Participants were followed through 31 December 2003, for a total of 17.3 years of follow-up. Incident cases of the first occurrence of primary lung cancer [*International Classification of Diseases for Oncology* (ICD-O-3) code C34] were identified by linkage of the full cohort to the Netherlands Cancer Registry and to the nationwide network and registry of histopathology and cytopathology (PALGA). A total of 3,355 incident cases of lung cancer [1,298 squamous-cell carcinomas (ICD-O-3 8050–8076), 573 small-cell carcinomas (ICD-O-3 8040–8045), 498 large-cell carcinomas (ICD-O-3 8012–8031, 8310), 737 adenocarcinomas (ICD-O-3 8140, 8211, 8230–8231, 8250–8260, 8323, 8480–8490), and 249 with other or unknown histological subtypes] were identified.

*Exposure assessment*. Each exposure metric was calculated based on only the baseline (1986) home address of each participant. The methods for calculating long-term average (1987–1996) exposures of NO_2_, black smoke (BS), and PM_2.5_ have been described in detail (Beelen et al. 2007, 2008). In brief, the regional, urban, and local contributions of each pollutant were determined and summed to obtain background concentrations (the sum of regional and urban contributions) or overall concentrations (the sum of the background and local contributions) for each participant. The regional contribution was predicted using inverse distance weighting of monitoring at regional background locations from the National Air Quality Monitoring Network (http://www.rivm.nl/en/Documents_and_publications/Scientific/Reports/1999/maart/The_Dutch_National_Air_Quality_Monitoring_Network_monitoring_program_in_1999?sp=cml2bXE9ZmFsc2U7c2VhcmNoYmFzZT01OTAxMDtyaXZtcT1mYWxzZTs=&pagenr=5902), whereas urban predictions were estimated using a land-use regression model including data from all regional and urban background monitoring sites and variables for population density and residential or industrial land use. The local contribution was estimated from land-use regressions incorporating monitoring data from field monitoring campaigns and a variety of traffic variables as predictors. Three measures of exposure to traffic were defined using a geographic information system (GIS) using a digital road network and traffic intensity information from 1986: *a*) an indicator for living near a major road, defined as within 100 m of a motorway or within 50 m of a local road with ≥ 10,000 vehicles per 24 hr, *b*) the traffic intensity in vehicles per 24 hr (mvh/24 hr) on the nearest road, and *c*) the sum of traffic intensity times road length within a 100-m buffer around the residential address in vehicles per 24 hr. We have previously shown that although the traffic intensities have increased during the follow-up period, data from different years were highly correlated, even over periods as long as 10 years (Beelen et al. 2007, 2008).

*Exposure validation data*. Details of the validation study have also been published previously (Van Roosbroeck et al. 2008a). Briefly, personal and near-home outdoor exposures to PM_2.5_ absorbance, NO_2_, and PM_2.5_ were collected for 48 hr up to five times from 47 adult nonsmoking participants living in Utrecht between November 2004 and July 2005. PM_2.5_ absorbance and BS are both surrogates of black carbon obtained by filter reflectance measurement but from different types of filters (Roorda-Knape et al. 1998). Approximately 50% lived near roads with a traffic intensity ≥ 10,000 mvh/24 hr, and 50% lived on on streets with < 5,000 mvh/24 hr, > 50 m from a road ≥ 10,000 mvh/24 hr, and > 400 m away from freeways with traffic intensities higher than 70,000 mvh/24 hr. We explored the utility of this validation study to correct our health effect estimates for the difference between personal and ambient measures of BS, NO_2_, and PM_2.5_.

*Statistical analysis*. Cox proportional hazards models were used to determine the associations of each measure of exposure to traffic or air pollution with risk of incident lung cancer overall or specific histological subtype. For continuous exposures, after assessing linearity using restricted cubic splines (Durrleman and Simon 1989; Govindarajulu et al. 2007) and performing log-likelihood tests to determine the best-fitting model, we calculated hazard ratios (HRs) and 95% CIs for an interquartile range increase (10 μg/m^3^ for BS and PM_2.5_, 30 μg/m^3^ for NO_2_, 10,000 mvh/24 hr for traffic intensity on the nearest road, and 335,000 mvh/24 hr for traffic intensity in a 100-m buffer). To account for the additional variance introduced by the case-cohort design, standard errors were estimated using the robust sandwich estimator (Lin and Wei 1989). We adjusted for a number of *a priori* potential confounders including age (as the time metric); sex; body mass index (BMI); cigarette, cigar, and pipe smoking status; number of cigarettes/cigars/pipes smoked on average; years of each type of tobacco use; home exposure to secondhand smoke; educational attainment; classification of the last occupation; and consumption of alcohol, fruits, vegetables, fish, and shellfish. We also adjusted all models for area-level indicators of socioeconomic status (SES) based on data from Statistics Netherlands: Percent of individuals below the 40th percentile and percent of individuals above the 80th percentile of the Dutch income distribution were calculated at both the neighborhood and “COROP area scale.” The COROP areas were defined in 1970 by the Dutch Coordination Commission for Regional Research Program to be a geographic region consisting of a city and the surrounding economic and social region. Missing indicator variables were created as needed for all variables. In sensitivity analyses, each *a priori* confounder (or group of confounders) was added to our basic models to determine if it (they) changed the association of any exposure on the risk of overall lung cancer by 10% (Greenland 1989). These confounders were then included in an alternate multivariable model to determine the sensitivity of our findings to our *a priori* selections. In sensitivity analyses to adjust our variance estimates for potential nonindependence among participants living in similar areas, we included random effects for each of the COROP areas in our multivariable models.

We performed stratified analyses by cigarette smoking status (current, former, never), overall tobacco use (current, former, never), and sex and created multiplicative interaction terms to assess effect modification. We also used multiplicative interaction terms to test effect modification by study follow-up period (original vs. extended). To test for heterogeneity in effect estimates across lung cancer subtypes, we used partial likelihood ratio tests from polytomous regressions using the publically available SUBTYPE macro (Kuchiba et al. 2014). A *p*-value of 0.05 was used to denote statistical significance.

*Measurement error adjustment*. We used the regression calibration method to adjust for bias due to exposure measurement error (Rosner et al. 1990; Spiegelman et al. 1997), using the publicly available BLINPLUS macro (Logan and Spiegelman 2012). First, we obtained the basic and multivariable adjusted HRs and 95% CIs as described above. Next, in the validation study, we regressed the measures of personal exposure on ambient exposure while controlling for age and sex. Then, measurement error–corrected point and interval estimates of the HRs were calculated by combining the uncorrected HRs from the Cox model with the validation study exposure regressions using a multivariate version of the following equation: β^ˆ^_1_ = β^ˆ^_1_*/γˆ_1_ where β^ˆ^_1_ is the measurement error–corrected effect estimate, β^ˆ^_1_* is the uncorrected effect estimate, and γˆ_1_ is the slope of the regression of personal exposure on exposure surrogate estimated in the validation study. The variance for the measurement error–corrected estimates incorporates the variance from estimating β_1_* in the main study, as well as from estimating γ_1_ in the validation study using the multivariate delta method.

As shown in previous simulation studies (Kuha 1994; Rosner et al. 1989, 1990; Spiegelman et al. 1997, 2001), regression calibration can be reliably performed when a number of assumptions have been satisfied. The assumptions include the following: *a*) The relationship between the personal and ambient exposure must be linear and homoscedastic, *b*) the associations between outcome and exposure must be linear on the scale of the link function used, *c*) the degree of measurement error is not severe, *d*) the measurement error is nondifferential, and *e*) the ambient exposure measure would not be associated with the outcome of interest if personal exposures were available. We examined the validity of the linearity assumptions using restricted cubic regression splines. Homoscedasticity in the validation study model was assessed by calculating the correlation between the predicted values and the absolute residuals from the linear regression models, and the statistical significance of deviations was assessed with the White test (White 1980). The magnitude of measurement error was examined by calculating β^ˆ^_1^2^_σˆ^2^, where σˆ^2^ is the residual variance from the regression of the personal exposures on the ambient exposures. Simulation studies have found that measurement error corrections are accurate when β_1_^2^σ^2^ < 0.5 (Kuha 1994). Nondifferential measurement error is reasonably assumed in this setting, where the exposure is measured prospectively and objectively, and participants subsequently followed for the occurrence of lung cancer. The fifth assumption is assumed to hold, because there is no reason to assume that ambient exposures would be associated with lung cancer independently of associations with personal exposures. In addition to the above assumptions, we must make the empirically unverifiable transportability assumption that the slope of the regression of the personal exposure on the ambient exposures found in the validation study would be similar to the one that would be found in the main study population. All data analyses were performed in SAS 9.3 (SAS Institute Inc.).

## Results

Cases were more likely to smoke cigarettes, cigars, and pipes than subcohort members, and were more exposed to secondhand smoke from a spouse (Table 1). They were also more likely to be male, to be less educated, and to work in blue-collar occupations. There was little difference in the measures of exposure and area-level SES between the cases and subcohort members, and the distributions of BMI and age were similar.

**Table 1:** Baseline (1986) characteristics of the lung cancer cases and the subcohort from the Netherlands Cohort Study on Diet and Cancer (*n *= 7,881).

Characteristic	Cases (*n *= 3,355)	Subcohort (*n *= 4,666)
Median (IQR)
Age (years)	62 (7)	61 (7)
Fruit and fruit preserves consumed (g/day)	106 (146)	145 (150)
Vegetables consumed (g/day)	165 (102)	175 (102)
Fish and shellfish consumed (g/day)	8 (20)	7 (20)
Percent neighborhood < 40th percentile of income	41 (11)	40 (10)
Percent neighborhood > 80th percentile of income	17 (12)	18 (13)
Percent COROP area < 40th percentile of income	41 (9)	41 (9)
Percent COROP area > 80th percentile of income	19 (5)	19 (5)
Average black smoke 1987–1996 (μg/m^3^)	16.7 (4.0)	16.6 (4.0)
Average NO_2_ 1987–1996 (μg/m^3^)	38.0 (11.0)	37.8 (11.1)
Average PM_2.5_ 1987–1996 (μg/m^3^)	28.3 (2.4)	28.3 (2.5)
Percent
Male	85.3	48.9
Marital status
Married	84.3	78.1
Single, divorced, widowed	15.6	21.5
Missing	0.1	0.4
Cigarette-smoking status
Never	6.8	36.3
Former	29.5	35.1
Current	63.8	28.6
Cigar-smoking status
Never	77.3	87.4
Former	6.6	5.4
Current	15.4	6.5
Pipe-smoking status
Never	87.3	92.5
Former	3.4	3.6
Current	8.2	3.1
Cigarette-smoking spouse
Never	41.3	30.8
Former	17.1	27.8
Current	34.2	31.6
NA or missing	7.4	9.9
Alcohol consumption (g/day)
< 0.4 (abstainer)	14.5	22.2
0.4–4	18.3	27.0
5–14	22.8	21.7
15–29	22.6	15.0
≥ 30	18.2	8.8
Missing	3.6	5.3
Educational attainment
Primary/lower vocational school	24.4	20.3
High school	55.0	51.5
Higher vocational or university	19.9	27.3
Missing	0.8	0.9
BMI (kg/m^2^)
< 20	3.7	3.5
20 to < 25	50.6	48.2
25 to < 30	38.2	38.3
≥ 30	4.0	6.4
Missing	3.6	3.6
Last occupation
Blue collar	36.7	26.7
Low white collar	12.6	15.8
White collar	20.7	19.8
Other	14.7	15.5
Last occupation ≥ 40 years ago	1.6	5.0
Never paid employment	1.4	6.5
Missing	12.4	10.7
Abbreviations: IQR, interquartile range; NA, not applicable.		

In age- and sex-adjusted models, HRs for all three pollutants and the measures of traffic exposure were above the null for associations with all lung cancer cases and with the specific histological subtypes (Table 2). There was no statistically significant evidence of heterogeneity across subtypes (all *p*-for-heterogeneity > 0.19). In models adjusted for our full set of *a priori* confounders, the HRs generally remained positive. All forms of tobacco use, educational attainment, marital status, occupation, diet, alcohol consumption, and neighborhood- and COROP-level SES were included in the parsimonious multivariable models, and results were similar to those from the *a priori* multivariable models (see Supplemental Material, Table S1). Although the random term for COROP area was statistically significant for many models (data not shown), the HRs from models accounting for potential clustering were similar to our main models (see Supplemental Material, Table S1). There was no evidence of effect modification by cigarette-smoking status, other tobacco use, sex, or follow-up period (*p*-values for interaction > 0.05; data not shown).

**Table 2:** Associations of increases in average black smoke, NO_2_, or PM_2.5_ exposures 1987–1996 or baseline address traffic measures with incident lung cancer 1986–2003 overall and by subtype.

Exposure	All lung cancer HR (95% CI)	Squamous-cell carcinoma HR (95% CI)	Small-cell carcinoma HR (95% CI)	Large-cell carcinoma HR (95% CI)	Adenocarcinoma HR (95% CI)
No. of cases	3,355	1,298	573	498	737
Black smoke (10 μg/m^3^)
Basic model^*a*^	1.23 (1.08, 1.40)	1.16 (0.96, 1.40)	1.24 (0.96, 1.60)	1.22 (0.94, 1.59)	1.42 (1.14, 1.78)
Multivariable model^*b*^	1.16 (1.02, 1.32)	1.14 (0.94, 1.38)	1.24 (0.95, 1.61)	1.22 (0.91, 1.62)	1.14 (0.90, 1.44)
NO_2_ (30 μg/m^3^)
Basic model^*a*^	1.24 (1.05, 1.47)	1.09 (0.86, 1.38)	1.21 (0.87, 1.69)	1.26 (0.89, 1.78)	1.65 (1.24, 2.21)
Multivariable model^*b*^	1.29 (1.08, 1.54)	1.24 (0.96, 1.61)	1.37 (0.95, 1.97)	1.33 (0.90, 1.97)	1.29 (0.93, 1.78)
PM_2.5_ (10 μg/m^3^)
Basic model^*a*^	1.12 (0.89, 1.40)	1.02 (0.73, 1.41)	1.11 (0.71, 1.72)	1.17 (0.73, 1.87)	1.44 (0.98, 2.11)
Multivariable model^*b*^	1.17 (0.93, 1.47)	1.15 (0.82, 1.61)	1.12 (0.71, 1.77)	1.37 (0.83, 2.26)	1.12 (0.74, 1.70)
Living near a major road
Basic model^*a*^	1.18 (0.96, 1.45)	1.15 (0.87, 1.54)	1.39 (0.96, 2.00)	1.28 (0.86, 1.91)	1.15 (0.81, 1.63)
Multivariable model^*c*^	1.12 (0.92, 1.37)	1.08 (0.80, 1.44)	1.40 (0.96, 2.02)	1.25 (0.83, 1.88)	1.05 (0.75, 1.47)
Traffic intensity on the nearest road (10,000 mvh/24 hr)
Basic model^*a*^	1.06 (0.96, 1.17)	1.07 (0.95, 1.21)	1.08 (0.89, 1.32)	1.03 (0.86, 1.23)	1.05 (0.92, 1.20)
Multivariable model^*c*^	1.02 (0.93, 1.12)	1.03 (0.91, 1.15)	1.06 (0.87, 1.29)	1.01 (0.84, 1.21)	0.99 (0.86, 1.14)
Traffic intensity in a 100-m buffer (335,000 mvh/24 hr)
Basic model^*a*^	1.15 (1.01, 1.31)	1.21 (1.01, 1.44)	1.13 (0.87, 1.46)	1.03 (0.79, 1.35)	1.20 (0.97, 1.49)
Multivariable model^*c*^	1.10 (0.97, 1.24)	1.17 (0.98, 1.39)	1.15 (0.89, 1.47)	0.98 (0.75, 1.29)	1.10 (0.89, 1.36)
^***a***^Adjusted for age and sex. ^***b***^Additionally adjusted for cigarette-, cigar-, and pipe-smoking status; years and amount of cigarette, cigar, and pipe smoking; secondhand smoke exposure; educational status; occupational status; marital status; BMI; alcohol consumption; intake of fruits, vegetables, and fish; and neighborhood- and COROP-level SES. ^***c***^Adjusted for all covariates in the default multivariable model plus regional and urban background black smoke.					

There was no evidence of deviation from linearity or evidence of deviation from homoscedasticity for any of the examined exposures in the validation data [Table 3, calculated with data from Van Roosbroeck et al. (2008a)]. Based on β_1_^2^σ^2^, the magnitude of measurement error was well within the bounds of the Kuha criterion [β_1_^2^σ^2^ < 0.5 (Kuha 1994)] for validity of regression calibration for BS (β_1_^2^σ^2^s of 0.008, 0.007, 0.011, 0.010, and 0.007 for all cases, squamous-cell carcinoma, small-cell carcinoma, large-cell carcinoma, and adenocarcinomas, respectively), and for PM_2.5_ (β_1_^2^σ^2^s of 0.316, 0.279, 0,224, 0.634, and 0.224 for all cases, squamous-cell carcinoma, small-cell carcinoma, large-cell carcinoma, and adenocarcinomas, respectively). However, the Kuha criterion was not satisfied for NO_2_ (β_1_^2^σ^2^s of 2.052, 1.753, 2.523, 2.281, and 2.033 for all cases, squamous-cell carcinoma, small-cell carcinoma, large-cell carcinoma, and adenocarcinomas, respectively). Therefore, any error corrections for NO_2_ would not be appropriate.

**Table 3:** Exposure information from the validation study (Van Roosbroeck et al. 2008a) available for measurement error correction.

Data	PM_2.5_ absorbance^*a*^ (10^–5^/m)	NO_2_ (μg/m^3^)	PM_2.5_ (μg/m^3^)
*n*	172	209	174
Measured personal exposure (mean ± SD)	1.71 ± 0.70	26.9 ± 11.3	16.8 ± 11.2
Measured ambient exposure (mean ± SD)	1.61 ± 0.63	32.0 ± 8.4	18.2 ± 10.0
Ratio of personal and ambient SDs	1.11	1.35	1.12
Correlation of personal and ambient exposures	0.78	0.04	0.45
Validation model *R*^2^	0.62	0.22	0.21
*p*-Value for test of heteroscedasticity	0.74	0.14	0.77
σ^2^	0.044	0.326	1.004
Deattenuation factor^*b*^	0.87	0.05	0.50
^***a***^PM_2.5_ absorbance was measured in the validation study and is used to adjust models for black smoke. ^***b***^The deattenuation factor is calculated by multiplying the ratio of the personal and ambient exposure standard deviations by the correlation between the personal and ambient measures.			

After adjustment for measurement error, the HRs for BS and PM_2.5_ were further from the null than the HRs before adjustment, with increases of 0–3.3% for BS and 9.7–37.2% for PM_2.5_ (Table 4). The magnitude of the percent increase in the width of the confidence intervals was generally an order of magnitude larger, with increases of 10.2–23.3% for BS and 108.0–216.8% for PM_2.5_.

**Table 4:** Measurement error–adjusted associations per interquartile range increase in black smoke or PM_2.5_ exposures on the risk of incident lung cancer 1986–2003 overall and by subtype.

Exposure	All cases HR (95% CI)^*a*^	Squamous-cell carcinoma HR (95% CI)^*a*^	Small-cell carcinoma HR (95% CI)^*a*^	Large-cell carcinoma HR (95% CI)^*a*^	Adenocarcinoma HR (95% CI)^*a*^
Black smoke (10 μg/m^3^)	1.19 (1.02, 1.39)	1.17 (0.93, 1.47)	1.28 (0.94, 1.75)	1.26 (0.90, 1.76)	1.17 (0.89, 1.54)
Percent increase in HR^*b*^	2.6	2.6	3.2	3.3	0.0
Percent increase in 95% CIs^*c*^	23.3	22.7	22.7	21.1	10.2
PM_2.5_ (10 μg/m^3^)	1.37 (0.86, 2.17)	1.32 (0.67, 2.61)	1.25 (0.50, 3.15)	1.88 (0.68, 5.21)	1.25 (0.54, 2.89)
Percent increase in HR^*b*^	17.1	14.8	11.6	37.2	9.7
Percent increase in 95% CIs^*c*^	142.6	145.6	150.0	216.8	108.0
^***a***^Multivariable model was adjusted for age and sex; cigarette-, cigar-, and pipe-smoking status; years and amount of cigarette, cigar, and pipe smoking; secondhand smoke exposure; educational status; occupational status; marital status; BMI; alcohol consumption; intake of fruits, vegetables, and fish; and neighborhood- and COROP-level SES. ^***b***^[(HR_multivariable_ – HR_measurement error_)/HR_multivariable_] × 100. ^***c***^{[(UCL_multivariable_ – LCL_multivariable_) – (UCL_measurement error_ – LCL_measurement error_)]/(UCL_multivariable_ – LCL_multivariable_)} × 100.					

## Discussion

In this extended follow-up of the NLCS, HRs were above the null for risks of overall and histologic subtype–specific lung cancer for exposures to BS, NO_2_, PM_2.5_, and with measures of traffic at the baseline address, even after adjustment for a number of lifestyle and dietary factors, and personal and area-level SES. Associations were positive for all histologic subtypes; however, there was no statistically significant heterogeneity observed. Adjustment for measurement error to account for the differences between personal and ambient exposures led to modest increases in the HRs for BS (0–3.3%) and moderate increases in the HRs for PM_2.5_, (9.7–37.2%), along with substantial widening of the confidence intervals (10.2–216.8%).

Adjustment for various aspects of measurement error has become more common in studies of air pollution in recent years. Several methods have been proposed to address the impact of potential errors induced due to the spatial modeling of exposure (Molitor et al. 2007; Sheppard et al. 2012; Szpiro and Paciorek 2013; Szpiro et al. 2011). Others have adjusted estimates of the effects of air pollution on some health end points for the differences between personal and ambient point exposures (Avery et al. 2010a, 2010b; Holliday et al. 2014). These authors used random-effects meta-analysis of literature-based reported correlations between personal and ambient exposures to impute personal exposures for the main study.

Although there was little evidence of effect modification by follow-up period, our results had HRs of greater magnitude and more were statistically significant compared with our previous findings in this cohort (Beelen et al. 2008). For example, in the present analysis, the HR for BS was 1.16 (95% CI: 1.02, 1.32, per 10 μg/m^3^), compared with an equivalent HR of 1.03 (95% CI: 0.78, 1.34) in our previous analysis. Additionally, we observed HRs > 1 with exposures to PM_2.5_ and NO_2_, which were not observed in the previous analysis. However, although we had previously observed differences in these associations by smoking status, we did not observe statistically significant differences by smoking status in the present analysis.

Most studies of PM_2.5_ on lung cancer risk have reported positive associations, even with a wide variety of approaches to exposure assessment, and a mix of incident and mortality studies (Cao et al. 2011; Carey et al. 2013; Cesaroni et al. 2013; Hart et al. 2011; Hystad et al. 2013; Jerrett et al. 2013; Katanoda et al. 2011; Krewski et al. 2009; Lepeule et al. 2012; Lipsett et al. 2011; McDonnell et al. 2000; Puett et al. 2014; Raaschou-Nielsen et al. 2013). Our measurement error–corrected and –uncorrected HRs for PM_2.5_ on overall lung cancer incidence are near the higher end of the distribution of results from previous studies (see Supplemental Material, Table S2). In a recent meta-analysis that included the estimate from our previous NLCS lung cancer analysis, the risk ratio for a 10-μg/m^3^ increase was estimated to be 1.09 (95% CI; 1.04, 1.14) (Hamra et al. 2014).

A large number of studies from around the world have also reported that NO_2_ exposures are positively associated with lung cancer risk (Abbey et al. 1999; Carey et al. 2013; Cesaroni et al. 2013; Filleul et al. 2005; Hart et al. 2011; Heinrich et al. 2013; Hystad et al. 2013; Jerrett et al. 2013; Katanoda et al. 2011; Krewski et al. 2009; Lipsett et al. 2011; Nyberg et al. 2000; Raaschou-Nielsen et al. 2013; Villeneuve et al. 2014; Yorifuji et al. 2013). Our HR of 1.29 (95% CI: 1.08, 1.54 for each 30-μg/m^3^ increase in NO_2_) is near the center of the distribution of findings from previous studies (see Supplemental Material, Table S3). As with PM_2.5_, positive associations have been reported based on a wide variety of study types from around the world, with a number of different approaches to exposure assessment.

To our knowledge, only two other population-based studies have explored the associations of BS or related measures with risk of lung cancer. In the French Pollution Atmospherique et Affections Respiratoires Chronique (PAARC) study, exposure to BS in seven French cities was associated with an increased risk of lung cancer (adjusted HR = 1.03; 95% CI: 0.92, 1.15 for each 10-μg/m^3^ increase) (Filleul et al. 2005). The multi-country European Study of Cohorts for Air Pollution Effects (ESCAPE) used PM_2.5_ absorbance as a marker of BS, and also observed positive associations (HR = 1.12; 95% CI: 0.88, 1.42, per 10^–5^/m increase) (Raaschou-Nielsen et al. 2013).

Results of studies examining the impact of roadway proximity on the risk of lung cancer risk have been more mixed. In addition to our previous analysis, a number of other studies have examined distance to roadway or traffic intensity as an exposure (Cesaroni et al. 2013; Hystad et al. 2013; Puett et al. 2014; Raaschou-Nielsen et al. 2011, 2013; Vineis et al. 2006). Similar to our findings, these studies have generally observed modest increases in lung cancer risk. Given the heterogeneity in methods and definitions, however, the different metrics are difficult to compare, and few studies have observed statistically significant results.

Although we observed HRs of different magnitudes for the different lung cancer subtypes we examined, there was no statistically significant heterogeneity among the subtypes. Differences of effect among subtypes are of great interest, but to date only a limited number of studies have examined histological subtype–specific effects. This interest in differences by subtype is motivated by differences in risk observed with exposures to cigarette smoking. For example, small-cell carcinoma, squamous-cell carcinoma, and adenocarcinomas have been the subtypes most closely associated with cigarette smoking (Boyle et al. 2010; Tse et al. 2009). Stronger associations with various pollutants have been observed for adenocarcinomas and squamous-cell carcinomas. Specifically, in ESCAPE, elevated HRs were observed in models of PM_2.5_ exposure restricted to these two subtypes when compared with models of all cases (Raaschou-Nielsen et al. 2013). In a case–control study in Canada, subtype-specific results for PM_2.5_ and NO_2_ were mixed, with a suggestion of a larger risk for adenocarcinomas compared with other subtypes (Hystad et al. 2013). Positive associations with exposures to PM were also observed for adenocarcinomas compared with all lung cancer cases in a study of U.S. nurses (Puett et al. 2014).

This study has several limitations. We used exposures based on the baseline home address as a proxy for actual exposures over time. However, a number of studies have also demonstrated that land-use regressions, such as the one used here, are quite robust to historical changes (Cesaroni et al. 2012; Eeftens et al. 2011; Gulliver et al. 2013). Our inability to incorporate changes in residence during the study period would have induced further exposure misclassification. Another limitation is that we were not able to adjust our analyses of NO_2_ (due to violations in the required assumptions) and the traffic proximity and volume measures (due to a lack of data in the validation study) for measurement error. The high β_1_^2^σ^2^ for NO_2_ is likely attributable to the presence of indoor sources or low air exchange rates, which have been consistently observed in other studies (Kousa et al. 2001; Lai et al. 2004; Rotko et al. 2001; Sahsuvaroglu et al. 2009; Zipprich et al. 2002). Given the differences in measurement error for PM_2.5_ and BS, it is not possible to determine the potential magnitude error that would be observed for NO_2_. We are also not able to quantify the impact of indoor sources of NO_2_ on lung cancer risk. Therefore, our NO_2_ associations should be treated with caution and interpreted only as the ambient effects of these exposures. Last, as with all studies, residual confounding is a concern. Our study was not able to update potential confounders, such as smoking or diet, after baseline, and we were missing information on potential confounders such as secondhand smoke and occupation for around 10% of the study participants.

Our validation study and measurement error approach also have some limitations. Information was available from only 45 individuals, with a few more than 200 individual sampling sessions. This limits our ability to examine personal characteristics that may impact the personal and ambient exposure relationships. We were not able to directly measure BS in the validation study, and instead measured PM_2.5_ absorbance, which is measured from another type of filter. However, these two measurements are highly correlated (*R*^2^ = 0.94) (Roorda-Knape et al. 1998), so this is unlikely to be a major source of error. There were also a number of differences between the population measured in the validation study and the individuals in the subcohort. For example, the validation study was composed of nonsmokers in a single metropolitian area of the Netherlands, and it was conducted after the NLCS follow-up. If there are substantially different relationships of personal to ambient exposure measures between the members of the validation study and NLCS, then the assumption of transportability would be violated, and it would not be appropriate to measurement error correct. The personal concentrations are affected by both indoor and outdoor sources. For studies on outdoor air pollution, it has been argued that personal exposure to outdoor- and indoor-generated particles should be considered separately (Wilson and Brauer 2006; Wilson et al. 2000). The correlation between outdoor exposure and the personal exposure to ambient origin pollution is the most relevant correlation, but difficult to assess. One method is to exclude the main indoor source from the study, as was done in the present validation study by excluding smokers.

This study also has major strengths. The long follow-up period and high rate of case ascertainment have provided us with a large number of cases with information on histological subtype. This allows us to examine the impact of a number of pollutants on subtype-specific risks, which to date has been possible in only a handful of studies. Our use of regression calibration to adjust for bias due to measurement error in predicted ambient pollutant concentrations in relation to personal exposure measurements, though imperfect, provides a sense of the level of underestimation in studies that are unable to perform this correction for measurement error bias.

In conclusion, in this large study based in the Netherlands, we observed an elevated risk of overall and histologic subtype–specific incident lung cancer with long-term exposure to BS, NO_2_, PM_2.5_, and with measures of traffic at the baseline address. The HRs increased after correction for measurement error, although the impact of the adjustment for measurement error varied between the two pollutants where adjustment was possible. Correction for measurement error also resulted in substantial losses in precision. These findings add support to a growing body of literature on the effects of air pollution on lung cancer, as well as to the recent classification of air pollution as a human carcinogen by IARC (Loomis et al. 2013).

## Supplemental Material

(298 KB) PDF

## References

[r1] Abbey DE, Nishino N, McDonnell WF, Burchette RJ, Knutsen SF, Lawrence Beeson W (1999). Long-term inhalable particles and other air pollutants related to mortality in nonsmokers.. Am J Respir Crit Care Med.

[r2] Allodji RS, Thiébaut AC, Leuraud K, Rage E, Henry S, Laurier D (2012). The performance of functional methods for correcting non-Gaussian measurement error within Poisson regression: corrected excess risk of lung cancer mortality in relation to radon exposure among French uranium miners.. Stat Med.

[r3] Armstrong BA. (2004). Exposure measurement error: consequences and design issues. In: Exposure Assessment in Occupational and Environmental Epidemiology (Nieuwenhuijsen MJ, ed.).

[r4] Armstrong BG (1990). The effects of measurement errors on relative risk regressions.. Am J Epidemiol.

[r5] Avery CL, Mills KT, Williams R, McGraw KA, Poole C, Smith RL (2010a). Estimating error in using ambient PM_2.5_ concentrations as proxies for personal exposures: a review.. Epidemiology.

[r6] Avery CL Mills KT Williams R McGraw KA Poole C Smith RL 2010b Estimating error in using residential outdoor PM_2.5_ concentrations as proxies for personal exposures: a meta-analysis. Environ Health Perspect 118 673 678; 10.1289/ehp.0901158 20075021 PMC2866684

[r7] Beelen R, Hoek G, Fischer P, van den Brandt PA, Brunekreef B (2007). Estimated long-term outdoor air pollution concentrations in a cohort study.. Atmos Environ.

[r8] Beelen R, Hoek G, van den Brandt PA, Goldbohm RA, Fischer P, Schouten LJ (2008). Long-term exposure to traffic-related air pollution and lung cancer risk.. Epidemiology.

[r9] Boyle P, Gray N, Henningford J, Seffrin J, Zatonski W. (2010). Tobacco: Science, Policy and Public Health. 2nd ed..

[r10] Cao J, Yang C, Li J, Chen R, Chen B, Gu D (2011). Association between long-term exposure to outdoor air pollution and mortality in China: a cohort study.. J Hazard Mater.

[r11] Carey IM, Atkinson RW, Kent AJ, van Staa T, Cook DG, Anderson HR (2013). Mortality associations with long-term exposure to outdoor air pollution in a national English cohort.. Am J Respir Crit Care Med.

[r12] Cesaroni G Badaloni C Gariazzo C Stafoggia M Sozzi R Davoli M 2013 Long-term exposure to urban air pollution and mortality in a cohort of more than a million adults in Rome. Environ Health Perspect 121 324 331; 10.1289/ehp.1205862 23308401 PMC3621202

[r13] Cesaroni G Porta D Badaloni C Stafoggia M Eeftens M Meliefste K 2012 Nitrogen dioxide levels estimated from land use regression models several years apart and association with mortality in a large cohort study. Environ Health 11 48; 10.1186/1476-069X-11-48 22808928 PMC3407745

[r14] Durrleman S, Simon R (1989). Flexible regression models with cubic splines.. Stat Med.

[r15] Eeftens M, Beelen R, Fischer P, Brunekreef B, Meliefste K, Hoek G (2011). Stability of measured and modelled spatial contrasts in NO_2_ over time.. Occup Environ Med.

[r16] Fearn T, Hill DC, Darby SC (2008). Measurement error in the explanatory variable of a binary regression: regression calibration and integrated conditional likelihood in studies of residential radon and lung cancer.. Stat Med.

[r17] Filleul L, Rondeau V, Vandentorren S, Le Moual N, Cantagrel A, Annesi-Maesano I (2005). Twenty five year mortality and air pollution: results from the French PAARC survey.. Occup Environ Med.

[r18] Govindarajulu US, Spiegelman D, Thurston SW, Ganguli B, Eisen EA (2007). Comparing smoothing techniques in Cox models for exposure–response relationships.. Stat Med.

[r19] Greenland S (1989). Modeling and variable selection in epidemiologic analysis.. Am J Public Health.

[r20] Gulliver J, de Hoogh K, Hansell A, Vienneau D (2013). Development and back-extrapolation of NO_2_ land use regression models for historic exposure assessment in Great Britain.. Environ Sci Technol.

[r21] Hamra GB Guha N Cohen A Laden F Raaschou-Nielsen O Samet JM 2014 Outdoor particulate matter exposure and lung cancer: a systematic review and meta-analysis. Environ Health Perspect 122 906 911; 10.1289/ehp.1408092 24911630 PMC4154221

[r22] Hart JE, Garshick E, Dockery DW, Smith TJ, Ryan L, Laden F (2011). Long-term ambient multipollutant exposures and mortality.. Am J Respir Crit Care Med.

[r23] Heid IM, Küchenhoff H, Miles J, Kreienbrock L, Wichmann HE (2004). Two dimensions of measurement error: classical and Berkson error in residential radon exposure assessment.. J Expo Anal Environ Epidemiol.

[r24] Heinrich J, Thiering E, Rzehak P, Krämer U, Hochadel M, Rauchfuss KM (2013). Long-term exposure to NO_2_ and PM_10_ and all-cause and cause-specific mortality in a prospective cohort of women.. Occup Environ Med.

[r25] Holliday KM, Avery CL, Poole C, McGraw K, Williams R, Liao D (2014). Estimating personal exposures from ambient air pollution measures: using meta-analysis to assess measurement error.. Epidemiology.

[r26] Horick N Weller E Milton DK Gold DR Li R Spiegelman D 2006 Home endotoxin exposure and wheeze in infants: correction for bias due to exposure measurement error. Environ Health Perspect 114 135 140; 10.1289/ehp.7981 16393671 PMC1332669

[r27] Hystad P, Demers PA, Johnson KC, Carpiano RM, Brauer M (2013). Long-term residential exposure to air pollution and lung cancer risk.. Epidemiology.

[r28] Jerrett M, Burnett RT, Beckerman BS, Turner MC, Krewski D, Thurston G (2013). Spatial analysis of air pollution and mortality in California.. Am J Respir Crit Care Med.

[r29] Katanoda K, Sobue T, Satoh H, Tajima K, Suzuki T, Nakatsuka H (2011). An association between long-term exposure to ambient air pollution and mortality from lung cancer and respiratory diseases in Japan.. J Epidemiol.

[r30] Keshaviah AP, Weller E, Spiegelman D (2003). Occupational exposure to methyl tertiary butyl ether in relation to key health symptom prevalence: the effect of measurement error correction.. Environmetrics.

[r31] Kousa A, Monn C, Rotko T, Alm S, Oglesby L, Jantunen M (2001). Personal exposures to NO_2_ in the *EXPOLIS*-study: relation to residential indoor, outdoor and workplace concentrations in Basel, Helsinki and Prague.. Atmos Environ.

[r32] Krewski D, Jerrett M, Burnett RT, Ma R, Hughes E, Shi Y (2009). Extended follow-up and spatial analysis of the American Cancer Society study linking particulate air pollution and mortality.. Res Rep Health Eff Inst.

[r33] Kuchiba A, Wang M, Spiegelman D. (2014). The SAS SUBTYPE Macro.. http://www.hsph.harvard.edu/donna-spiegelman/software/subtype/.

[r34] Kuha J (1994). Corrections for exposure measurement error in logistic regression models with an application to nutritional data.. Stat Med.

[r35] Lai HK, Kendall M, Ferrier H, Lindup I, Alm S, Hänninen O (2004). Personal exposures and microenvironment concentrations of PM_2.5_, VOC, NO_2_ and CO in Oxford, UK.. Atmos Environ.

[r36] Lepeule J Laden F Dockery D Schwartz J 2012 Chronic exposure to fine particles and mortality: an extended follow-up of the Harvard Six Cities study from 1974 to 2009. Environ Health Perspect 120 965 970; 10.1289/ehp.1104660 22456598 PMC3404667

[r37] Li R, Weller E, Dockery DW, Neas LM, Spiegelman D (2006). Association of indoor nitrogen dioxide with respiratory symptoms in children: application of measurement error correction techniques to utilize data from multiple surrogates.. J Expo Sci Environ Epidemiol.

[r38] Lin DY, Wei LJ (1989). The robust inference for the Cox proportional hazards model.. J Am Stat Assoc.

[r39] Lipsett MJ, Ostro BD, Reynolds P, Goldberg D, Hertz A, Jerrett M (2011). Long-term exposure to air pollution and cardiorespiratory disease in the California Teachers Study cohort.. Am J Respir Crit Care Med.

[r40] Logan R, Spiegelman D. (2012). The SAS %BLINPLUS Macro.. http://www.hsph.harvard.edu/donna-spiegelman/software/blinplus-macro/.

[r41] Loomis D, Grosse Y, Lauby-Secretan B, El Ghissassi F, Bouvard V, Benbrahim-Tallaa L (2013). The carcinogenicity of outdoor air pollution.. Lancet Oncol.

[r42] McDonnell WF, Nishino-Ishikawa N, Petersen FF, Chen LH, Abbey DE (2000). Relationships of mortality with the fine and coarse fractions of long-term ambient PM_10_ concentrations in nonsmokers.. J Expo Anal Environ Epidemiol.

[r43] Molitor J Jerrett M Chang CC Molitor NT Gauderman J Berhane K 2007 Assessing uncertainty in spatial exposure models for air pollution health effects assessment. Environ Health Perspect 115 1147 1153; 10.1289/ehp.9849 17687440 PMC1940074

[r44] Nyberg F, Gustavsson P, Jarup L, Bellander T, Berglind N, Jakobsson R (2000). Urban air pollution and lung cancer in Stockholm.. Epidemiology.

[r45] Puett RC Hart JE Yanosky JD Spiegelman D Wang M Fisher JA 2014 Particulate matter air pollution exposure, distance to road, and incident lung cancer in the Nurses’ Health Study cohort. Environ Health Perspect 122 926 932; 10.1289/ehp.1307490 24911062 PMC4154215

[r46] Raaschou-Nielsen O, Andersen ZJ, Beelen R, Samoli E, Stafoggia M, Weinmayr G (2013). Air pollution and lung cancer incidence in 17 European cohorts: prospective analyses from the European Study of Cohorts for Air Pollution Effects (ESCAPE).. Lancet Oncol.

[r47] Raaschou-Nielsen O Andersen ZJ Hvidberg M Jensen SS Ketzel M Sørensen M 2011 Lung cancer incidence and long-term exposure to air pollution from traffic. Environ Health Perspect 119 860 865; 10.1289/ehp.1002353 21227886 PMC3114823

[r48] Raaschou-Nielsen O, Bak H, Sørensen M, Jensen SS, Ketzel M, Hvidberg M (2010). Air pollution from traffic and risk for lung cancer in three Danish cohorts.. Cancer Epidemiol Biomarkers Prev.

[r49] Roorda-Knape MC, Janssen NAH, de Hartog JJ, van Vliet PHN, Harssema H, Brunekreef B (1998). Air pollution from traffic in city districts near major roadways.. Atmos Environ.

[r50] Rosner B, Spiegelman D, Willett WC (1990). Correction of logistic regression relative risk estimates and confidence intervals for measurement error: the case of multiple covariates measured with error.. Am J Epidemiol.

[r51] Rosner B, Willett WC, Spiegelman D (1989). Correction of logistic regression relative risk estimates and confidence intervals for systematic within-person measurement error.. Stat Med.

[r52] Rotko T, Kousa A, Alm S, Jantunen M (2001). Exposures to nitrogen dioxide in *EXPOLIS*-Helsinki: microenvironment, behavioral and sociodemographic factors.. J Expo Sci Environ Epidemiol.

[r53] Sahsuvaroglu T, Su JG, Brook J, Burnett R, Loeb M, Jerrett M (2009). Predicting personal nitrogen dioxide exposure in an elderly population: integrating residential indoor and outdoor measurements, fixed-site ambient pollution concentrations, modeled pollutant levels, and time–activity patterns.. J Toxicol Environ Health A.

[r54] Sheppard L, Burnett RT, Szpiro AA, Kim SY, Jerrett M, Pope CA (2012). Confounding and exposure measurement error in air pollution epidemiology.. Air Qual Atmos Health.

[r55] Spiegelman D (2010). Approaches to uncertainty in exposure assessment in environmental epidemiology.. Annu Rev Public Health.

[r56] Spiegelman D, Carroll RJ, Kipnis V (2001). Efficient regression calibration for logistic regression in main study/internal validation study designs with an imperfect reference instrument.. Stat Med.

[r57] Spiegelman D, McDermott A, Rosner B (1997). Regression calibration method for correcting measurement-error bias in nutritional epidemiology.. Am J Clin Nutr.

[r58] Szpiro AA, Paciorek CJ (2013). Measurement error in two-stage analyses, with application to air pollution epidemiology.. Environmetrics.

[r59] Szpiro AA, Sheppard L, Lumley T (2011). Efficient measurement error correction with spatially misaligned data.. Biostatistics.

[r60] Tse LA, Mang OW, Yu IT, Wu F, Au JS, Law SC (2009). Cigarette smoking and changing trends of lung cancer incidence by histological subtype among Chinese male population.. Lung Cancer.

[r61] van den Brandt PA, Goldbohm RA, van ‘t Veer P, Volovics A, Hermus RJ, Sturmans F (1990). A large-scale prospective cohort study on diet and cancer in the Netherlands.. J Clin Epidemiol.

[r62] Van Roosbroeck S, Hoek G, Meliefste K, Janssen NA, Brunekreef B (2008a). Validity of residential traffic intensity as an estimate of long-term personal exposure to traffic-related air pollution among adults.. Environ Sci Technol.

[r63] Van Roosbroeck S, Li R, Hoek G, Lebret E, Brunekreef B, Spiegelman D (2008b). Traffic-related outdoor air pollution and respiratory symptoms in children: the impact of adjustment for exposure measurement error.. Epidemiology.

[r64] Villeneuve PJ, Jerrett M, Brenner D, Su J, Chen H, McLaughlin JR (2014). A case-control study of long-term exposure to ambient volatile organic compounds and lung cancer in Toronto, Ontario, Canada.. Am J Epidemiol.

[r65] Villeneuve PJ, Jerrett M, Su J, Burnett RT, Chen H, Brook J (2013). A cohort study of intra-urban variations in volatile organic compounds and mortality, Toronto, Canada.. Environ Pollut.

[r66] Vineis P, Hoek G, Krzyzanowski M, Vigna-Taglianti F, Veglia F, Airoldi L (2006). Air pollution and risk of lung cancer in a prospective study in Europe.. Int J Cancer.

[r67] White H (1980). A heteroskedasticity-consistent covariance matrix estimator and a direct test for heteroskedasticity.. Econometrica.

[r68] Wilson WE, Brauer M (2006). Estimation of ambient and non-ambient components of particulate matter exposure from a personal monitoring panel study.. J Expo Sci Environ Epidemiol.

[r69] Wilson WE, Mage DT, Grant LD (2000). Estimating separately personal exposure to ambient and nonambient particulate matter for epidemiology and risk assessment: why and how.. J Air Waste Manag Assoc.

[r70] Yorifuji T, Kashima S, Tsuda T, Ishikawa-Takata K, Ohta T, Tsuruta K (2013). Long-term exposure to traffic-related air pollution and the risk of death from hemorrhagic stroke and lung cancer in Shizuoka, Japan.. Sci Total Environ.

[r71] Zhukovsky M, Onishchenko A, Varaksin A, Vasilyev A (2011). The influence of radon measurement errors on the uncertainties of epidemiological case–control studies.. Radiat Prot Dosimetry.

[r72] Zipprich JL, Harris SA, Fox JC, Borzelleca JF (2002). An analysis of factors that influence personal exposure to nitrogen oxides in residents of Richmond, Virginia.. J Expo Sci Environ Epidemiol.

