# Supplementary material for: Long-Term Ambient Residential Traffic–Related Exposures and Measurement Error–Adjusted Risk of Incident Lung Cancer in the Netherlands Cohort Study on Diet and Cancer
Source: Environ Health Perspect. 2015 Mar 27;123(9):860–6. doi: 10.1289/ehp.1408762 (PMC4559954; doi:10.1289/ehp.1408762)
Supplement: (298 KB) PDF [file ehp.1408762.s001.acco.pdf]

Environ Health Perspect
doi: 10.1289/ehp.1408762

Note to Readers: ***EHP*** **strives to ensure that all journal content is accessible to all readers. However, some figures and Supplemental Material published in *EHP* articles may not conform to 508 standards due to the complexity of the information being presented. If you need assistance accessing journal content, please contact ehp508@niehs.nih.gov. Our staff will work with you to assess and meet your accessibility needs within 3 working days.**

Supplemental Material

Long-Term Ambient Residential Traffic–Related Exposures and Measurement Error–Adjusted Risk of Incident Lung Cancer in the Netherlands Cohort Study on Diet and Cancer

Jaime E. Hart, Donna Spiegelman, Rob Beelen, Gerard Hoek, Bert Brunekreef, Leo J. Schouten, and Piet van den Brandt

Table of Contents

Table S1: Associations of Increases in Black Smoke, NO_2, or $PM_{2.5}$ Exposures or Baseline Address Traffic Measures with Incident Lung Cancer 1986-2003 Overall and by Subtype; Sensitivity Models Adjusted only for Covariates that Change Effect Estimates by 10% or More (Parsimonious) and Multivariable Models Additionally Adjusted for Clustering by COROP Area.

Table S2: Literature Summary of Associations of $PM_{2.5}$ and Lung-Cancer Calculated for each 10 $\mu g/m^3$ Increase and Presented in Increasing Hazard Ratio Order.

Table S3: Literature Summary of Associations of NO_2 and Lung-Cancer per 30 $\mu g/m^3$ Increase and Presented in Increasing Hazard Ratio Order.

References

Table S1: Associations of Increases in Black Smoke, NO_2, or $PM_{2.5}$ Exposures or Baseline Address Traffic Measures with Incident Lung Cancer 1986-2003 Overall and by Subtype; Sensitivity Models Adjusted only for Covariates that Change Effect Estimates by 10% or More (Parsimonious) and Multivariable Models Additionally Adjusted for Clustering by COROP Area.

**Exposure**	**All Lung Cancer HR (95%CI)**	**Squamous Cell Carcinoma HR (95%CI)**	**Small Cell Carcinoma HR (95%CI)**	**Large Cell Carcinoma HR (95%CI)**	**Adenocarcinoma HR (95%CI)**
Number of cases	3,355	1,298	573	498	737
Black smoke (10 μg/m^3)					
Parsimonious[a]	1.16 (1.02, 1.32)	1.13 (0.93, 1.37)	1.20 (0.92, 1.56)	1.22 (0.91, 1.61)	1.17 (0.93, 1.47)
Multivariable model + clustering[b]	1.13 (1.00, 1.28)	1.17 (0.96, 1.42)	1.24 (0.96, 1.60)	1.06 (0.75, 1.51)	1.16 (1.02, 1.32)
NO_2 (30 μg/m^3)					
Parsimonious[a]	1.28 (1.07, 1.53)	1.21 (0.94, 1.57)	1.29 (0.90, 1.86)	1.31 (0.88, 1.93)	1.33 (0.96, 1.83)
Multivariable model[b]	1.27 (1.07, 1.51)	1.37 (1.03, 1.83)	1.37 (0.97, 1.93)	1.10 (0.66, 1.83)	1.28 (1.07, 1.53)
$PM_{2.5}$ (10 μg/m^3)					
Parsimonious[a]	1.16 (0.92, 1.45)	1.13 (0.81, 1.59)	1.08 (0.68, 1.70)	1.32 (0.80, 2.18)	1.14 (0.75, 1.72)
Multivariable model + clustering[b]	1.14 (0.90, 1.44)	1.22 (0.84, 1.77)	1.10 (0.69, 1.75)	1.20 (0.62, 2.32)	1.16 (0.92, 1.45)
Living near a major road					
Parsimonious[a]	1.13 (0.93, 1.38)	1.08 (0.81, 1.45)	1.38 (0.95, 2.01)	1.30 (0.87, 1.94)	1.06 (0.76, 1.48)
Multivariable model + clustering[c]	1.13 (0.96, 1.31)	1.08 (0.84, 1.40)	1.39 (0.99, 1.97)	1.36 (0.93, 2.00)	1.13 (0.93, 1.38)
Traffic intensity on the nearest road (10,000 mvh/24h)					
Parsimonious[a]	1.02 (0.93, 1.12)	1.03 (0.92, 1.16)	1.07 (0.87, 1.30)	1.02 (0.85, 1.21)	0.99 (0.86, 1.14)
Multivariable model + clustering[c]	1.02 (0.94, 1.10)	1.03 (0.91, 1.16)	1.06 (0.89, 1.25)	1.02 (0.85, 1.23)	1.02 (0.93, 1.12)
Traffic intensity in a 100-m buffer (335,000 mvh/24 h)					
Parsimonious[a]	1.10 (0.98, 1.25)	1.17 (0.98, 1.39)	1.15 (0.90, 1.47)	1.00 (0.76, 1.31)	1.10 (0.89, 1.37)
Multivariable model + clustering[c]	1.09 (0.99, 1.21)	1.16 (0.99, 1.36)	1.15 (0.90, 1.46)	1.06 (0.80, 1.39)	1.10 (0.98, 1.25)

[a]Adjusted for age and sex, cigarette, cigar, and pipe smoking status, years and amount of cigarette, cigar, and pipe smoking, marital status, educational status, occupational status, alcohol consumption, intake of fruits, vegetables, and fish, and neighborhood- and COROP-level SES. [b]Adjusted for age, sex, clustering by COROP, cigarette, cigar, and pipe smoking status, years and amount of cigarette, cigar, and pipe smoking, marital status, secondhand smoke exposure, educational status, occupational status, BMI, alcohol consumption, intake of fruits, vegetables, and fish, and neighborhood- and COROP-level SES. [c]Adjusted for all covariates in the default multivariable model plus regional and urban background black smoke.

Table S2: Literature Summary of Associations of $PM_{2.5}$ and Lung-Cancer Calculated for each 10 $\mu g/m^3$ Increase and Presented in Increasing Hazard Ratio Order.

Study	RR (95%CI)	Study Location
(Lipsett et al. 2011)	0.95 (0.70,1.28)	United States
(Cao et al. 2011)	1.03 (1.00,1.07)	China
(Cesaroni et al. 2013)	1.05 (1.01,1.10)	Italy
(Puett et al. 2014)	1.06 (0.91,1.25)	United States
(Krewski et al. 2009)	1.08 (1.03,1.14)	United States
(Carey et al. 2013)	1.11 (0.85,1.43)	England
(Jerrett et al. 2013)	1.12 (0.92,1.37)	United States
Current study – multivariable models	1.17 (0.93,1.47)	Netherlands
(Hart et al. 2011)	1.17 (0.93,1.48)	United States
(Katanoda et al. 2011)	1.24 (1.12,1.37)	Japan
(Hystad et al. 2013)	1.29 (0.95,1.76)	Canada
Current Study – measurement error adjusted	1.37 (0.86,2.17)	Netherlands
(Lepeule et al. 2012)	1.37 (1.07,1.75)	United States
(McDonnell et al. 2000)	1.39 (0.79,2.46)	United States
(Raaschou-Nielsen et al. 2013)	1.39 (0.92,2.10)	Europe

The effect estimates from (Naess et al. 2007) could not be converted to units of 10 $\mu g/m^3$ and are therefore not shown.

Table S3: Literature Summary of Associations of NO_2 and Lung-Cancer per 30 μg/m^3 Increase and Presented in Increasing Hazard Ratio Order.

Study	RR (95%CI)	Study Location
(Raaschou-Nielsen et al. 2013)	0.97 (0.80, 1.17)	Europe
(Krewski et al. 2009)	0.98 (0.95, 1.02)	United States
(Lipsett et al. 2011)	1.00 (0.64, 1.56)	United States
(Cesaroni et al. 2013)	1.12 (1.06, 1.19)	Italy
(Hart et al. 2011)	1.15 (0.92, 1.43)	United States
(Carey et al. 2013)	1.18 (1.00, 1.39)	England
(Hystad et al. 2013)	1.18 (1.00, 1.40)	Canada
(Katanoda et al. 2011)	1.28 (1.16, 1.42)	Japan
Current Study	1.29 (1.08, 1.54)	Netherlands
(Nyberg et al. 2000)	1.33 (0.91, 1.94)	Sweden
(Jerrett et al. 2013)	1.50 (1.08, 2.09)	United States
(Abbey et al. 1999), men	1.62 (0.94, 2.79)	United States
(Yorifuji et al. 2013)	1.73 (1.09, 2.73)	Japan
(Heinrich et al. 2013)	2.03 (0.86, 4.83)	Germany
(Abbey et al. 1999), women	2.30 (1.12, 4.73)	United States
(Filleul et al. 2005)	3.24 (1.16, 9.08)	France
(Villeneuve et al. 2014)	4.67(1.78, 12.24)	Canada

The effect estimates from (Naess et al. 2007) could not be converted to units of 30 μg/m^3 and are therefore not included.

References

Abbey DE, Nishino N, McDonnell WF, Burchette RJ, Knutsen SF, Lawrence Beeson W, et al. 1999. Long-term inhalable particles and other air pollutants related to mortality in nonsmokers. Am J Respir Crit Care Med 159:373-382.

Cao J, Yang C, Li J, Chen R, Chen B, Gu D, et al. 2011. Association between long-term exposure to outdoor air pollution and mortality in China: A cohort study. J Hazard Mater 186:1594-1600.

Carey IM, Atkinson RW, Kent AJ, van Staa T, Cook DG, Anderson HR. 2013. Mortality associations with long-term exposure to outdoor air pollution in a national English cohort. American journal of respiratory and critical care medicine 187:1226-1233.

Cesaroni G, Badaloni C, Gariazzo C, Stafoggia M, Sozzi R, Davoli M, et al. 2013. Long-term exposure to urban air pollution and mortality in a cohort of more than a million adults in Rome. Environmental Health Perspectives 121:324-331.

Filleul L, Rondeau V, Vandentorren S, Le Moual N, Cantagrel A, Annesi-Maesano I, et al. 2005. Twenty five year mortality and air pollution: Results from the French PAARC survey. Occup Environ Med 62:453-460.

Hart JE, Garshick E, Dockery DW, Smith TJ, Ryan L, Laden F. 2011. Long-term ambient multipollutant exposures and mortality. Am J Respir Crit Care Med 183:73-78.

Heinrich J, Thiering E, Rzehak P, Kramer U, Hochadel M, Rauchfuss KM, et al. 2013. Long-term exposure to NO_2 and PM_{10} and all-cause and cause-specific mortality in a prospective cohort of women. Occup Environ Med 70:179-186.

Hystad P, Demers PA, Johnson KC, Carpiano RM, Brauer M. 2013. Long-term residential exposure to air pollution and lung cancer risk. Epidemiology 24:762-772.

Jerrett M, Burnett RT, Beckerman BS, Turner MC, Krewski D, Thurston G, et al. 2013. Spatial analysis of air pollution and mortality in California. American Journal of Respiratory and Critical Care Medicine 188:593-599.

Katanoda K, Sobue T, Satoh H, Tajima K, Suzuki T, Nakatsuka H, et al. 2011. An association between long-term exposure to ambient air pollution and mortality from lung cancer and respiratory diseases in Japan. J Epidemiol 21:132-143.

Krewski D, Jerrett M, Burnett RT, Ma R, Hughes E, Shi Y, et al. 2009. Extended follow-up and spatial analysis of the American Cancer Society study linking particulate air pollution and mortality. Res Rep Health Eff Inst:5-114; discussion 115-136.

Lepeule J, Laden F, Dockery D, Schwartz J. 2012. Chronic exposure to fine particles and mortality: An extended follow-up of the Harvard Six Cities study from 1974 to 2009. Environ Health Perspect 120:965-970.

Lipsett MJ, Ostro BD, Reynolds P, Goldberg D, Hertz A, Jerrett M, et al. 2011. Long-term exposure to air pollution and cardiorespiratory disease in the California Teachers Study cohort. Am J Respir Crit Care Med 184:828-835.

McDonnell WF, Nishino-Ishikawa N, Petersen FF, Chen LH, Abbey DE. 2000. Relationships of mortality with the fine and coarse fractions of long-term ambient PM_{10} concentrations in nonsmokers. Journal of exposure analysis and environmental epidemiology 10:427-436.

Naess O, Nafstad P, Aamodt G, Claussen B, Rosland P. 2007. Relation between concentration of air pollution and cause-specific mortality: Four-year exposures to nitrogen dioxide and particulate matter pollutants in 470 neighborhoods in Oslo, Norway. American Journal of Epidemiology 165:435-443.

Nyberg F, Gustavsson P, Jarup L, Bellander T, Berglind N, Jakobsson R, et al. 2000. Urban air pollution and lung cancer in Stockholm. Epidemiology 11:487-495.

Puett RC, Hart JE, Yanosky JD, Spiegelman D, Wang M, Fisher JA, et al. 2014. Particulate matter air pollution exposure, distance to road, and incident lung cancer in the Nurses' Health Study cohort. Environmental Health Perspectives 122:926-932.

Raaschou-Nielsen O, Andersen ZJ, Beelen R, Samoli E, Stafoggia M, Weinmayr G, et al. 2013. Air pollution and lung cancer incidence in 17 european cohorts: Prospective analyses from the European Study of Cohorts for Air Pollution Effects (ESCAPE). Lancet Oncol 14:813-822.

Villeneuve PJ, Jerrett M, Brenner D, Su J, Chen H, McLaughlin JR. 2014. A case-control study of long-term exposure to ambient volatile organic compounds and lung cancer in Toronto, Ontario, Canada. American Journal of Epidemiology 179:443-451.

Yorifuji T, Kashima S, Tsuda T, Ishikawa-Takata K, Ohta T, Tsuruta K, et al. 2013. Long-term exposure to traffic-related air pollution and the risk of death from hemorrhagic stroke and lung cancer in Shizuoka, Japan. The Science of the total environment 443:397-402.
